# Circadian rhythm impacts preclinical FDG-PET quantification in the brain, but not in xenograft tumors

**DOI:** 10.1038/s41598-020-62532-8

**Published:** 2020-03-27

**Authors:** Marcel A. Krueger, Carsten Calaminus, Julia Schmitt, Bernd J. Pichler

**Affiliations:** Werner Siemens Imaging Center, Department of Preclinical Imaging, University of Tuebingen, Tuebingen, Germany

**Keywords:** Positron-emission tomography, Cancer imaging

## Abstract

The inner clock of biological organisms plays a pivotal role and has strong effects on metabolic processes such as glucose consumption. Since the commonly used positron emission tomography (PET) tracer ^18^F-flourodeoxygucose (FDG) is a glucose analogue, it is not surprising that the FDG distribution in mice and humans has been shown to succumb to daily rhythms. In preclinical studies, the circadian rhythm of animals is often not considered, and studies are performed at different times of day. Only a few studies have analyzed the effect of the circadian rhythm on FDG uptake in mice, and none of these studies included human tumor xenografts. Therefore, it is not known how strongly a preclinical tumor study is influenced by the time of day. In this work, the effect of the circadian rhythm on FDG uptake in human tumor xenografts and other organs was analyzed. CD1 nu/nu mice were kept for three weeks under a 12 h light/12 h dark rhythm and then injected s.c. with PC3 or A431 tumor cells. When the tumors had reached an appropriate volume, FDG-PET scans were performed on different animal groups (n = 4–5) every 4 h over a time period from 8 A.M. to 8 P.M. Tracer uptake in the tumors and in other organs was determined based on the PET scans and biodistribution studies. The standardized uptake value and %injected dose/cc of the tumors remained constant over the whole observed time period, and no statistically significant differences were determined according to the PET analysis. In the brain, we found a small but statistically significant increase from noon to 4 P.M., which led to a decrease in the tumor-to-brain ratio. No evidence for an effect of the circadian rhythm on FDG uptake could be found in subcutaneous tumors, however, in brain studies the circadian rhythm needs to be considered.

## Introduction

Due to the daily changes in the light-dark environment on earth, many organisms, as basic as cyanobacteria, have developed a circadian rhythm to adapt to these periodic changes^1,2^. In mammals, the suprachiasmatic nucleus (SCN) plays a pivotal role in maintaining this rhythm. In this small area of the hypothalamus, many single-cell oscillators are synchronized by external factors such as light and food intake^2,3^. The SCN sends periodic signals to different peripheral clocks in tissues such as muscle, liver, and adipose tissue^2^. The circadian rhythm affects a variety of physiological processes in the body, including sleep-wake cycles, body temperature, digestion, metabolism and secretion of corticosterone and insulin, which are well known to affect glucose homeostasis^4,5^.

The circadian rhythm also seems to play a role in the development and treatment of cancer. Patients suffering from metastatic breast cancer with an abnormal cortisol rhythm were shown to have a worse outcome than those with a normal cortisol rhythm^6^. Chronomodulated chemotherapy was demonstrated to increase the tolerability of chemotherapy for patients, but the sensitivity of the tumors was also affected^7–9^. Interestingly, it was shown that transplanted tumors had faster growth kinetics in mice with an ablated SCN^10^ and that tumor growth kinetics in mice are strongly affected by the circadian rhythm^11^. Together, these data underline the importance of the circadian rhythm on tumor growth and physiology in patients and preclinical tumor models.

Positron emission tomography (PET) is a frequently applied noninvasive imaging technology with growing importance in a wide range of different diseases such as cancer, coronary heart disease, and brain injury^12,13^. In oncology, the glucose analogue ^18^F-flourodeoxygucose (FDG) is by far the most frequently applied PET tracer in clinical practice as well as in clinical and preclinical research. FDG is transported into cells via glucose transporters (GLUTs) and is subsequently phosphorylated inside the cell. Since further metabolism of FDG in the glycolytic pathway is not possible, FDG is trapped in the cytoplasm^14,15^.

The effect of the circadian rhythm on glucose metabolism is well known^16^. Several hormones, such as insulin, glucagon and corticosterone, are regulated in a circadian manner, and blood glucose levels are altered throughout the day in mice and humans^16^. In mouse studies, FDG uptake has been investigated in brown adipose tissue (BAT), heart and brain, and a clear circadian regulation could be observed^13,17^.

Preclinical tumor studies can easily become time consuming when either large or many groups of mice are used or when dynamic PET scans are performed, especially if only one PET scanner is available. Furthermore, inconsistent tracer production schedules or deliveries might lead to varying starting times for the studies. Especially in longitudinal studies, this might be a considerable problem if not all single experiments are performed at the same time each day. However, in preclinical tumor studies applying PET, the effects of the circadian rhythm on FDG tracer uptake have not been analyzed. Therefore, we performed a standard FDG-PET study at several time points throughout a time span from 8 A.M. to 8 P.M. in nude mice bearing s.c. PC3 prostate cancer tumors or s.c. A431 epidermal tumors as examples of commonly used tumor models.

## Material and Methods

### Cells and tumor cell injection

PC3 and A431 tumor cells were obtained from ATCC (Wesel, Germany). Both cell lines were grown in a humidified atmosphere at 37 °C containing 5% CO_2_ in RPMI 1640 medium supplemented with 10% FCS, 100 U/mL penicillin and 100 mg/L streptomycin (all obtained from Biochrom, Berlin, Germany). To obtain tumor xenografts, 5 mio. PC3 or 10 mio. A431 cells were injected s.c. in the right flank of CD1 nu/nu mice in 200 µL NaCl solution (Braun, Melsungen, Germany) 8 or 7 days before the PET scans, respectively. One day before the PET scans, the animals were randomized and separated into 4 groups of 5 mice each.

### Animals and animal housing

Six-week-old female CD1 nu/nu mice were purchased from Charles River (Sulzfeld, Germany) and housed with a 12 h light/12 h dark rhythm with lights on at 7 A.M. and lights off at 7 P.M. and access to water and food *ad libitum* for three weeks before the tumor cell injections and until the start of the PET measurements. All applicable institutional and/or national guidelines for the care and use of animals were followed and the experiments were approved by the responsible local authorities (Regierungspraesidium Tuebingen).

### FDG-PET scans

All animals were starved for 6 h prior to the FDG injection. For injections, the animals were anesthetized with 1.5% isoflurane (Abbott, Wiesbaden, Germany) evaporated in oxygen at a flow rate of 0.5 L/min. Immediately before the injection of FDG, the blood glucose levels were determined by retro-orbital blood draw with a Hemocue 201 (Radiometer GmbH, Willich, Germany), and body weights of the mice were measured. In total, ~13 MBq FDG dissolved in 150 µL NaCl solution was injected i.v., and the animals were kept under isoflurane narcosis for 55 min. postinjection. All animals were warmed during the uptake period. PET scans were performed on an Inveon dedicated small-animal PET scanner (Siemens Preclinical Solutions, Knoxville, USA) for 10 min. while the mice were still under isoflurane narcosis. According to our standard protocol for mouse PET imaging, no attenuation and scatter correction were applied. Reconstruction was performed in Inveon Acquisition Workplace 1.5.0.28 with OSEM2D with four iterations. The reconstructed voxel size was 0.776 × 0.776 × 0.796 mm^3^.

### Biodistribution studies

Immediately after the PET scan, the animals were killed, and several organs were dissected, weighed and analyzed in a gamma counter (Wizard Wallac 1480, PerkinElmer Inc., Waltham, MA, USA). Subsequently the %ID/g of each organ was calculated.

### Image analysis

The images were analyzed in Inveon Research Workplace (Siemens Preclinical Solutions, Erlangen, Germany). The tumors and other organs were identified in the PET images, and regions of interest (ROIs) were drawn. Volumes of interest (VOIs) were calculated from the ROIs in Inveon Research Workplace. Tumor volumes, standardized uptake values (SUV) and %ID/cc of all analyzed tissues were determined.

### Statistics

A one-way ANOVA followed by a Tukey test was performed with Prism Version 7.03 (GraphPad Software, La Jolla, CA, USA). Significantly different values are indicated with * if p ≤ 0.05, with ** if p ≤ 0.01 and with *** if p ≤ 0.001.

## Results

### FDG-PET analysis of xenografts and other organs

**Figure 1:**
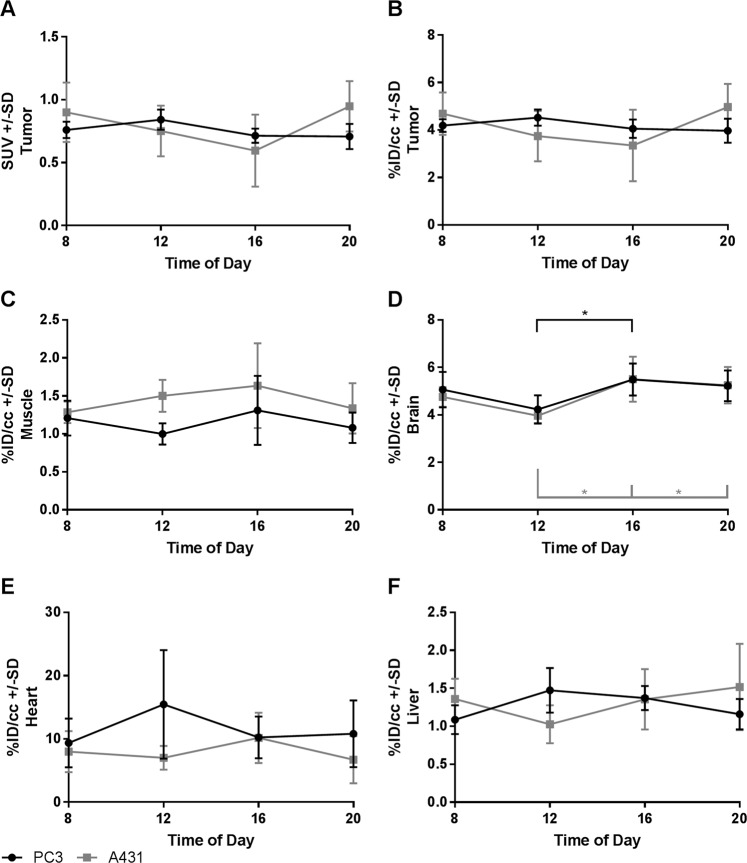
SUV (**A**) and %ID/cc (**B**) for the tumors and %ID/cc for the muscle (**C**), brain (**D**), heart (**E**) and liver (**F**); n = 4 or 5 for each data point.

To investigate the effect of the circadian rhythm on FDG uptake in human tumor xenografts and several organs, we performed FDG-PET scans with PC3 or A431 tumor-bearing mice at different times of the day and subsequently sacrificed the animals for biodistribution studies. The highest measured mean value in the PC3 tumor xenografts was an SUV of 0.84, which corresponds to a %ID/cc of 4.2 at noon. The lowest mean SUV value was 0.71, and the lowest %ID/cc was 4.0 at 8 P.M. (Fig. 1A,B). However, these changes were not statistically significant. In A431 tumors the SUV ranged from 0.6 to 0.95 at 4 P.M. and 8 P.M. respectively, corresponding to a %ID/cc of 3.4 and 5.0. Also in this tumor model no significant changes could be observed. The muscle showed no statistically significant fluctuations within groups with the same tumors. The highest uptake was measured at 4 P.M. with a %ID/cc of 1.6 in A431 tumor bearing mice, and the lowest uptake was found at noon with a %ID/cc of 1.0 in PC3 tumor bearing mice (Fig. 1C). In the brain, we observed a significant increase in the %ID/cc from noon to 4 P. M in both groups (*P* = 0.039 for PC3 and *P* = 0.01 for A431), and the %ID/cc ranged from ~4 at noon to 5.5 at 4 P.M. In the A431 tumor bearing mice a statistically significant difference could also be observed between the noon and 8 P.M. measurements (Fig. 1D). For the heart and liver, no statistically significant changes could be observed. In the heart mean values ranged between 6.7 and 15.5%ID/cc and in the liver values ranged from 1.0 to 1.5%ID/cc (Fig. 1E,F).

### Biodistribution studies

**Figure 2:**
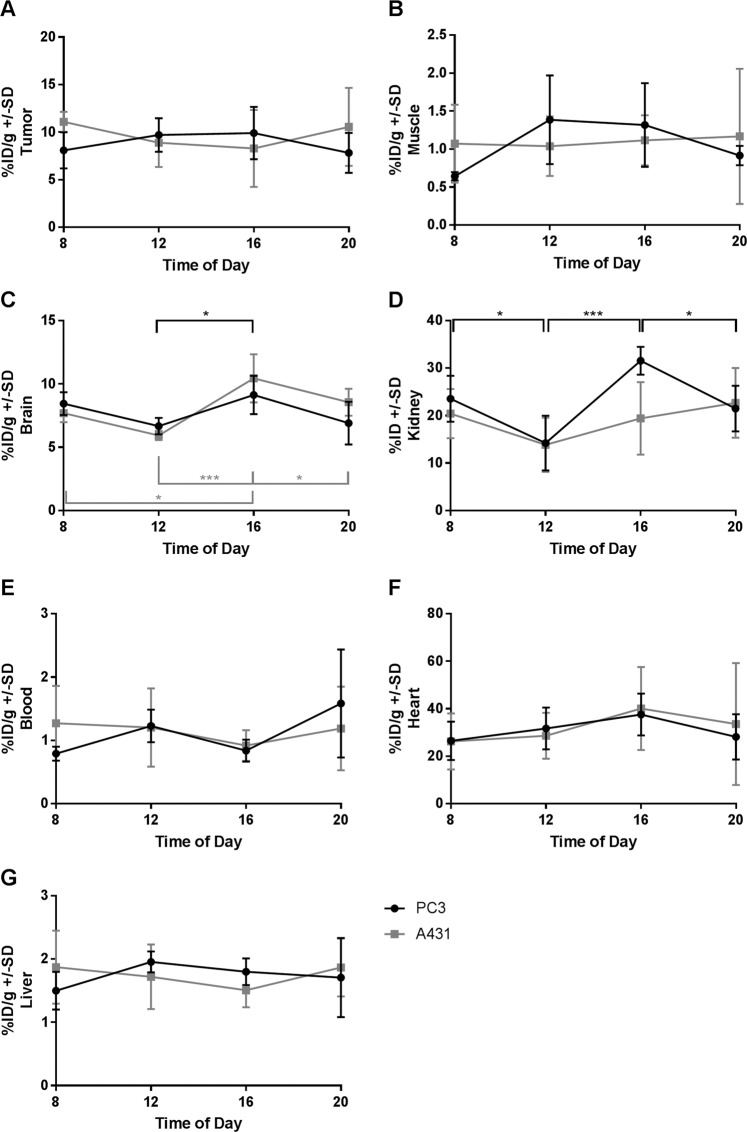
Gamma counter results of the %ID/g in the following isolated organs: tumor (**A**), muscle (**B**), brain (**C**), kidney (**D**), blood (**E**), heart (**F**) and liver (**G**); n = 4 or 5 for each data point.

In the *ex vivo* biodistribution study, the maximum FDG uptake in the tumor xenografts was reached at 4 P.M. with 9.9%ID/g for PC3 tumors and at 8 A.M. with 11.1%ID/g for A431 tumors, and the minimum in PC3 tumors was found at 8 P.M. with 7.8%ID/g and at 4 P.M. with 8.3%ID/cc in A431 tumors. However, in agreement with the *in vivo* quantification of the tumor uptake, this variability was not found to be significant for both tumor models (Fig. 2A). We also analyzed the FDG uptake in the muscle, brain, kidney, blood, heart and liver. In the muscle of PC3 tumor bearing mice uptake ranged between 0.6%ID/g at 8 A.M. and 1.4%ID/g at noon, with high standard deviations for the measurements at noon and 4 P.M. However, none of these fluctuations were statistically significant (Fig. 2B). In A431 bearing animals the uptake ranged only from 1.0 to 1.2%ID/g, again without statistically significant differences. In the brain, we observed mean values from 5.9 to 10.4%ID/g. The minimum was reached at noon, and the maximum was reached at 4 P.M. In accordance with the PET data, in PC3 tumor bearing animals a significant difference was found between the noon and 4 P.M. time points (*P* = 0.037), while in A431 tumor bearing animals a statistically significant difference was also found between the noon and 8 P.M. time point (*P* = 0.049) and the 8 A.M. and 4 P.M. time point (*P* = 0.016) (*P* = 0.001 for noon vs 4 P.M.) (Fig. 2C). The strongest circadian changes could be identified in the kidney, with mean values in PC3 tumor bearing mice from 14.2 to 31.6%ID/g, a peak FDG uptake at 4 P.M. and the lowest uptake at noon (Fig. 2D). In A431 tumor bearing mice values ranged between 14.2 and 22.7%ID/cc at noon and 8 P.M. respectively. In the blood, heart and liver, no significant differences between any of the analyzed time points in both tumor groups were found (Fig. 2E–G).

### Tumor-to-muscle and tumor-to-brain ratios

**Figure 3:**
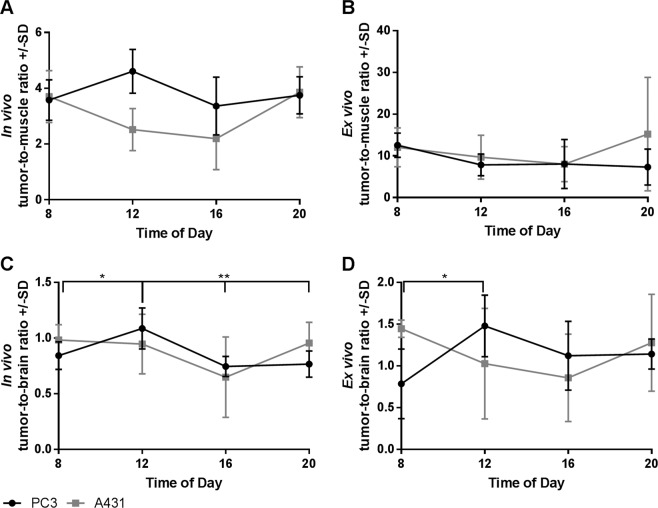
*In vivo* tumor-to-muscle and tumor-to-brain ratios (**A,C**) and the *ex vivo* tumor-to-muscle and tumor-to-brain ratios (**B,D**); n = 4 or 5 for each data point.

*In vivo*, the tumor-to-muscle ratio showed no statistically significant changes. The minimum for PC3 tumors was found at 4 P.M. with a ratio of 3.4, and the maximum was found at noon with a ratio of 4.6 (Fig. 3A). In A431 tumors the lowest tumor-to-muscle ratio was found at 4 P.M. with 4.2 and the highest at 8 P.M. with 3.9. The *ex vivo* analysis of the tumor-to-muscle ratio also showed no statistically significant differences. However, the maximum for PC3 tumors was found at 8 A.M. with a value of 12.5, and the minimum was found at 8 P.M. with a value of 7.3. In A431 tumors values ranged from 8.0 to 15.2 at 4 P.M. and 8 P.M. respectively (Fig. 3B).

As a consequence of the stable %ID/cc in the PC3 tumors and the variations in the %ID/cc in the brain, we observed changes in the tumor-to-brain ratio *in vivo* (Fig. 3C). For PC3 tumors the maximum was found at noon, and the minimum was found at 4 P.M. The tumor-to-brain ratio ranged from 0.7 to 1.1, and the measurement at noon was significantly different from that at all other time points analyzed. In A431 tumors the tumor-to-brain ratio ranged from 0.6 to 1.0 at 4 P.M. and 8 A.M. respectively. For A431 tumors no significant differences in the tumor-to-brain ratio could be observed. The *ex vivo* data qualitatively reflect the *in vivo* data; however, the ratio for PC3 tumors at noon was only significantly different from the 8 A.M. ratio (*P* = 0.034), and the values ranged between 0.8 and 1.5 (Fig. 3D). In A431 tumors the ratio ranged between 0.9 and 1.4 between 4 P.M. and 8 A.M. respectively, without any significant difference.

### Blood glucose levels

**Figure 4:**
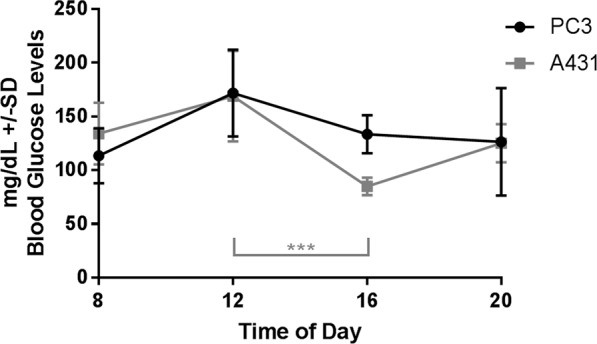
Blood glucose levels; n = 4 or 5 for each data point.

The blood glucose level varied over the examined time period with a peak at 12 P.M., and statistically significant differences in the A431 tumor bearing mice between noon and 4 P.M. could be detected (*P* = 0.001). The mean values at all time points ranged from 85–173 mg/dL (Fig. 4).

## Discussion

Our experiments show that the FDG uptake in subcutaneous PC3 and A431 tumors remained stable throughout the day. No major alterations were observed in the PET scans or in the *ex vivo* gamma counter biodistribution data. Additionally, for muscle, which is a frequently used reference tissue, no significant alterations were observed, and therefore, the tumor-to-muscle ratio appeared stable over the observed time period in both tumor models. In contrast, the uptake in the brain tissue had statistically significant changes during the analyzed time period. Due to these changes in the brain, we also observed statistically significant changes in the tumor-to-brain ratio of PC3 tumors. These findings are important to show that in our experimental setup, the circadian rhythm did not significantly affect the results of the tumor-related measurements, such as SUV, %ID/cc and tumor-to-muscle ratio, which are frequently used parameters in oncological studies. However, we can confirm the results from van der Veen *et al*., who showed that in brain studies, the circadian rhythm can affect tracer uptake^13^.

In our study, we saw that the regulation of FDG uptake in the brain had a peak in the afternoon during the analyzed time period. However, van der Veen *et al*. observed the highest FDG uptake at night, which was not included in our study since we only included time points that are realistic for performing oncological FDG-PET studies in our lab. In addition, the animals in the study from van der Veen *et al*. were not fasted, and the FDG uptake was measured in animals that were awake during uptake, which is in contrast to the methods of our study. Fasting, and especially isoflurane narcosis, is expected to have a major effect on brain activity and thus on metabolism and energy consumption. Therefore, a direct comparison of the two studies is difficult. However, neurology studies using FDG should be performed within a short time period to avoid effects from the circadian rhythm. In addition animals could be monitored with automated techniques as described by Fisher *et al*. to asses sleep/wake behavior of mice with digital video tracking to ensure similar phases of the circadian rhythm at the time of the PET scan^18^. Other techniques like EEG or EMG monitoring could also be applied, but are invasive and more expensive.

Based on the effects of fasting and isoflurane, a different FDG uptake protocol might also lead to a circadian regulation of FDG uptake in tumor xenografts. It is possible that the use of isoflurane during FDG uptake and fasting can dampen the circadian rhythm, and with other protocols, the tumor-related values might also be affected by the circadian rhythm. However, in this study, we only used the state-of-the-art set-up for tumor studies that is frequently used in many labs, *i.e*., fasted animals and FDG uptake under isoflurane narcosis^19^.

In addition to biological, anesthesia-related and animal handling-related effects, quantification variations caused by the circadian rhythm can add-up with effects triggered by imaging workflow-specific quantification errors such as attenuation, partial volume effects or scatter^20^.

For the heart and liver, the PET analysis did not reveal any significant changes in FDG uptake. This was also confirmed by the *ex vivo* biodistribution study. Additionally, in the blood, we did not find any significant changes in the %ID/g. However, the kidney showed a very strong circadian regulation of FDG uptake with mean values ranging from 13.8 to 31.6%ID/g, which was somewhat unexpected, and further experiments are required to understand this effect.

In PC3 tumor bearing animals the analysis of the muscle uptake from the biodistribution study and the PET scans show contradictory results. The PET analysis revealed a maximum uptake at 4 P.M. and a minimum at noon; in contrast, the minimum in the biodistribution study was at 8 A.M., and the maximum was found at noon. This could be explained by the fact that for the biodistribution study, we selected the muscle of the right hind leg, while in the PET analysis, we used the muscle of the right shoulder to avoid bladder artifacts.

Interestingly, looking at the mean uptake values and the pattern of the uptake at different times of the day in the brain, this appears to be very similar in PC3 and A431 bearing mice, which were measured as independent groups. This indicates that the measured values are stable and do not significantly vary from one experiment to the other, when animals are measured at the same time of day. For the tumors such comparison cannot be made, due to the different physiology of PC3 and A431 tumors. Here it would be necessary to scan the same tumors on different days. However, the results of such experiment would be weakened by the fact that tumors are constantly growing and changing.

In this study, we only examined two subcutaneous human xenograft tumor models and did not find effects of the circadian rhythm on tumor FDG uptake. However, it is not clear if these results can be generalized to other xenograft and syngeneic tumor models, such as CT26 in Balb/c mice, or endogenous tumor models, such as Rip1Tag2 and PymT mice. Eventually, if the circadian rhythm is affecting FDG uptake in a specific tumor model needs to be validated in every tumor model individually since a general assumption cannot be made. Furthermore, the uptake protocol needs to be taken into account.

## Conclusion

We show here that there is no effect of the circadian rhythm on the FDG uptake of PC3 and A431 tumors during a time period from 8 A.M. to 8 P.M. and also that the tumor-to-muscle ratio appears to be stable. However, we could observe significant changes in FDG uptake of the brain, and this resulted in significant variations in the tumor-to-brain ratios of PC3 tumors. Therefore, the circadian rhythm does not seem to affect studies with subcutaneous tumors; however, in brain studies, the circadian rhythm needs to be taken into account during the study planning process.
